# Annotations

**Published:** 1903-01-03

**Authors:** 


### ANNOTATIONS.

### London's New Pathologist.

A CONSIDERABLE flutter has lately been produced among certain general practitioners in the south of London by the action of Mr. Troutbeck, coroner for the south-western district, in employing Dr. Ludwig Freyberger to perform post mortem examinations instead of calling upon the medical officer who had attended the case before death. It is evident that the coroner in so doing did but accept the nominee of the County Council, but it is held by the aggrieved practitioners, that according to the strict letter of the law the coroner has no power, when a qualified medical practitioner has been in attendance, to summon for the purpose any other than this practitioner except in certain well-defined cases. Nevertheless, we can but feel that a pathological investigation, such as is involved in the making of a post-mortem examination, is something very decidedly out of the ordinary run of work of the general practitioner, and something which, if there is anything in specialism at all, ought certainly to fall into the hands of a specialist. In every London hospital of any standing the post mortem examinations are made by a special officer, who is, indeed, a most important personage, and we think that in regard to the very serious medico legal work which so often follows a post-mortem ordered by the coroner, the same kind of special skill ought to be available in this case also. Whether the County Council acted in a seemly manner in appointing Dr. Freyberger on the grounds given by the chairman of the Public Control Committee, is a matter as to which we will not at the present express any opinion.

### The University of London Election.

THE resignation by Sir Michael Foster of his seat in Parliament, as representing the University of London, will necessitate an election by that body, and among the candidates is a medical graduate who has done much good work for London and for London hospitals, and whose claims, if on these grounds alone, deserve every consideration by such of our readers as are members of the University. We refer to Sir William J. Collins. THE HOSPITAL can take no cognisance of political opinions, and so far as the address issued by Sir William refers to the major game of politics, as played on the arena at St. Stephen's, we say nothing. It is sufficiently obvious, however, that the representative of such an electorate as the University of London ought to be one who will make his voice heard and his influence felt in the House of Commons, and of this we may assuredly feel certain that if elected Sir William Collins will not be a nonentity. From his constant activity and almost constant success in whatever he has undertaken, and from the fact that he has for many years been a member of the Senate of the University, his address will undoubtedly appeal strongly to a large number of the electors, while another large number will probably be influenced by the part he has played in advancing scientific work and in encouraging the judicious support of the voluntary hospital system. He is a member of the Council of King Edward's Hospital Fund, in the deliberations of which body he has always taken an active part; he is hon. secretary of the League of Mercy, the success of which is good proof of his powers of adaptability; for many years he has been a member of the London County Council, and he has been a most successful chairman of that body. A matter in regard to which scientific London should always feel grateful to him is the active part which he then took in establishing a research laboratory for pathology at Claybury. Turning to his more distinctly professional position, he is surgeon to the Royal Eye Hospital and to the London Temperance Hospital, his steady and successful professional work at the latter of these institutions having done much to save it from being regarded as a mere fad, and to hold it to its proper position as a demonstration of the non-necessity of alcohol in the treatment of disease. There are many no doubt who will differ from Sir William Collins on many subjects, but all will agree that he is a strong man, and that he has been able in a striking degree to leave his mark on each thing that he has touched.

### Two Ways of Looking at an Oyster.

As is now well known a serious outbreak of typhoid fever has occurred at Portsmouth, Winchester, and Southampton, which has been traced with every semblance of probability, if not certainty, to the consumption of oysters derived from a source which has long been known to be liable to contamination with sewage. Whether, in view of the many sources of infection to which each sufferer may conceivably have been exposed, it will ever be possible to bring forward demonstrable proof that these particular oysters, infected in this particular way, were the cause of the mischief is beside the question. The point of importance, the thing which shows how scandalous is the condition of affairs which is allowed to go on from year to year in regard to oysters, is that, notwithstanding that the whole matter was investigated and reported upon years ago, and that typhoid infection has long been well known to be transmissible to man by oysters which have been laid in sewage-contaminated water, their sale still goes on. It is when we come to the problem, What is to be done? that we see how very differently the public and the trade regard the question of the oyster. It appears that a meeting of representatives of the trade, held some time ago at Fishmongers' Hall, recommended that a deputation should wait upon the Local Government Board to urge the necessity of immediate legislation with a view of prohibiting the discharge of any sewage into any river, estuary, or the sea on any part of the coast. In other words they ask that sanitary authorities all round the coast are to be put to enormous expense in dealing with their sewage in order that a few oyster growers may continue to fatten oysters in most unsuitable places, merely because they are handy for the markets and convenient to the traders. A far more reasonable view would seem to be that taken by the Southampton Health Committee, who have resolved to urge on the Local Government Board the necessity of legislation vesting in local authorities the complete control of all oyster beds or ponds within their respective jurisdictions, and giving them power to regulate and prohibit the sale of oysters therefrom.

